# Association of Plasma Level of TNF-Related Apoptosis-Inducing Ligand with Severity and Outcome of Sepsis

**DOI:** 10.3390/jcm9061661

**Published:** 2020-06-01

**Authors:** Hongseok Yoo, Jin Young Lee, Junseon Park, Jeong Hoon Yang, Gee Young Suh, Kyeongman Jeon

**Affiliations:** 1 Division of Pulmonary and Critical Care Medicine, Department of Medicine, Samsung Medical Center, Sungkyunkwan University School of Medicine, Seoul 06351, Korea; hs.yoo@skku.edu (H.Y.); suhgy@skku.edu (G.Y.S.); 2 Department of Critical Care Medicine, Samsung Medical Center, Sungkyunkwan University School of Medicine, Seoul 06351, Korea; yenayein@gmail.com (J.Y.L.); study13.park@samsung.com (J.P.); jhysmc@gmail.com (J.H.Y.); 3 Division of Cardiology, Department of Medicine, Samsung Medical Center, Sungkyunkwan University School of Medicine, Seoul 06351, Korea

**Keywords:** biomarker, necroptosis, RIPK3 protein, sepsis, TNF-related apoptosis-inducing ligand

## Abstract

Recent studies have suggested that TNF-related apoptosis-inducing ligand (TRAIL) is associated with mortality in sepsis, possibly through necroptosis. The objective of this study was to analyze the association between the plasma level of TRAIL and sepsis severity and outcomes. Furthermore, the plasma level of TRAIL was compared to that of receptor-interacting protein kinase-3 (RIPK3), a key executor of necroptosis, to identify any correlation between TRAIL and necroptosis. Plasma levels of TRAIL and RIPK3 from consecutively enrolled critically ill patients were measured by ELISA. Of 190 study patients, 59 (31.1%) and 84 (44.2%) patients were diagnosed with sepsis and septic shock, respectively. There was a trend of decreased plasma level of TRAIL across the control, sepsis, and septic shock groups. For 143 patients with sepsis, patients with low plasma TRAIL were more likely to have septic shock and higher SAPS3 and SOFA scores. However, no difference in 28-day and 90-day mortalities was observed between the two groups. The plasma level of TRAIL was inversely associated with RIPK3 in patients with sepsis. Plasma levels of TRAIL increased over time on days three and seven, and were inversely associated with sepsis severity and RIPK3 level, but not with mortality.

## 1. Introduction

Sepsis is a life-threatening organ dysfunction caused by a dysregulated host response to infection [1] that may culminate in organ failure and death. Despite continuous efforts to understand and improve outcomes of sepsis, it remains a commonly fatal disease [2,3,4]. In addition, the current definition of sepsis identifies a heterogeneous population of individuals with diverse patterns of immune response, organ dysfunction, and clinical outcomes [1]. Therefore, early diagnosis, precise stratification of severity, and accurate outcome prediction are critical in managing patients with sepsis. Various biomarkers, including C-reactive protein and procalcitonin, have been investigated to date [5,6]; however, no marker has demonstrated sufficient discriminatory power [7].

Tumor necrosis factor (TNF)-related apoptosis-inducing ligand (TRAIL) is a cytokine and member of the TNF superfamily. TRAIL initiates apoptosis of transformed cells or tumor cells by binding to death receptors (DR) 4 or 5 [8] and functions as an immune response regulator in sepsis [8,9]. More recently, however, it was discovered that TRAIL also functions as a trigger for necroptosis, a specific form of programmed cell necrosis characterized by its caspase-independent activation and release of damage-associated molecular patterns (DAMPs) which convey highly proinflammatory properties [10,11]. In a recent study, plasma TRAIL was related to poor outcomes in patients with sepsis [12]. A subsequent multicenter study suggested a relationship between the plasma level of TRAIL with necroptosis in sepsis; however, its association with mortality demonstrated a contradictory result [13]. Therefore, further studies are necessary to clarify the relationship between plasma TRAIL and predicting outcomes of patients with sepsis. In this study, we analyzed the association between the plasma level of TRAIL and severity and outcomes of sepsis. We also compared the plasma level of TRAIL to that of receptor-interacting protein kinase-3 (RIPK3), a well-known necroptosis mediator [14], to identify the correlation between TRAIL and necroptosis.

## 2. Materials and Methods

### 2.1. Study Design and Registry

This was a prospective observational study of the Samsung Medical Center Registry of Critical Illness (SMC RoCI), which is an on-going single-center prospective registry of the Samsung Medical Center (1989-bed, university affiliated, tertiary referral hospital in Seoul, South Korea) initiated in April 2014 for the purpose of establishing a human sample repository and developing new biological markers for critical illness [15]. The study was approved by the institutional review board of Samsung Medical Center. Written informed consent was obtained from patients or their legally authorized representative prior to enrollment.

### 2.2. Study Patients

Critically ill adult (≥19 years old) patients admitted to the medical intensive care unit (ICU) of Samsung Medical Center were considered eligible for inclusion in the registry. Exclusion criteria were as follows: (1) cognitive impairment, (2) inability to provide informed consent, (3) ICU admission for a simple procedure or postsurgical care, (4) transfer from other hospitals, (5) end-of-life decision or admission to facilitate comfort care, (6) hemoglobin < 8 g/dL upon admission or persistent bleeding, and (7) discharge within 24 h of admission to ICU. Screening and enrollment were completed within 24 h of ICU admission. Patients registered between April 2014 and December 2016 were included in the analysis. Some clinical data and RIPK3 levels from patients enrolled until August 2016 were reported in a previous study [15].

### 2.3. Data Collection

A trained study coordinator used hospital records for each patient to prepare a standardized case report form. Clinical data consisting of patient demographics, reason for ICU admission, severity of illness scoring, and laboratory data were obtained at the time of enrollment. Illness severity was assessed by the Acute Physiology and Chronic Health Evaluation II (APACH II) [16], Simplified Acute Physiology Score 3 (SAPS 3) [17], and Sequential Organ Failure Assessment (SOFA) scores [18]. The Revised Trauma Score was used to determine the severity of trauma patients [19]. The primary outcome was 28-day mortality. Secondary outcomes were in-hospital and 90-day mortality.

Sepsis was defined according to the third International Consensus Definitions for Sepsis and Septic Shock (Sepsis-3) [1]. Since enrollment for the registry began in April 2014, patients enrolled before release of the new definition were reclassified. Patients in the registry who did not meet the definition of either sepsis or septic shock were defined as controls. 

### 2.4. Measurement of Plasma TRAIL and RIPK3

Along with clinical data, 19 mL of whole blood was drawn from each patient within 48 h of study enrollment. When possible, additional blood samples were collected at day 3 (15 mL) and day 7 (15 mL). Blood samples were centrifuged within 4 h of collection. Plasma was separated and stored at −80 °C until further analysis. Plasma TRAIL level was measured from stored aliquots using commercially available TRAIL Human ELISA kits according to the manufacturer’s recommendations (R & D systems, Minneapolis, MN, USA). Plasma RIPK3 level was measured using a commercially available ELISA kit as per the manufacturer’s recommendations (CUSABIO, Houston, TX, USA) [15].

### 2.5. Statistical Analysis

Data are presented as numbers (percentages) for categorical variables, and as the median and interquartile range (IQR, 25th–75th percentiles) for continuous variables. Categorical variables were compared using the Chi-square test or Fisher’s exact test, while continuous variables were compared using the Mann–Whitney *U* test. Differences in plasma TRAIL level across control, sepsis, and septic shock groups were assessed with the Kruskal–Wallis test. Baseline characteristics, clinical status at ICU admission, illness severity, and mortality were compared between patients with low and high TRAIL level divided by the median level of plasma TRAIL. To evaluate the association between plasma TRAIL level and patient outcomes, a Kaplan–Meier curve was used to determine the 90-day survival curves according to plasma TRAIL. These were then compared using the log-rank test. Linear regression was applied to assess the association between plasma levels of TRAIL and RIPK3. The Friedman test was performed to determine differences among serial levels of TRAIL. The Wilcoxon signed rank test with Bonferroni correction was used to compare plasma TRAIL levels between days 0 and 3 and between days 3 and 7. 

All tests were two-sided, and a *p* value < 0.05 was considered significant. Data were analyzed using IBM SPSS Statistics 20.0 (IBM, Chicago, IL, USA).

## 3. Results

**Figure 1:**
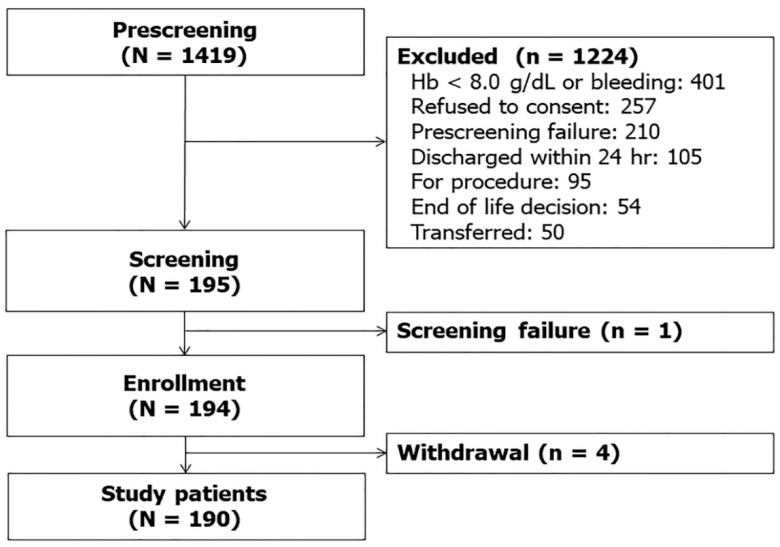
Study flow diagram.

Over the study period, 1419 patients were admitted to the ICU. After excluding 1224 patients who met the exclusion criteria and one patient who missed screening, 194 patients were enrolled in the registry. Since four patients withdrew their consent, 190 patients were finally included in the analysis (Figure 1).

The baseline characteristics of 190 patients at the time of ICU admission are summarized in Table 1. The median age was 64 (IQR, 54–73) years, and 121 (63.7%) patients were male. Of 62 (32.6%) patients with cancer, 32 patients had hematologic malignancy. The reason for ICU admission was sepsis in 59 (31.1%) patients and septic shock in 84 (44.2%) patients. For 47 patients of the control group, the most common cause of ICU admission was pulmonary edema in nine patients, followed by airway stenosis in six, interstitial lung disease in five, multiple trauma in five, and acute exacerbation of chronic obstructive pulmonary disease or asthma in four patients. The median revised trauma score for five patients with multiple trauma was 13.26 (IQR, 7.19–22.15). No patients in the control group were with infection. Ninety-five (50.0%) patients were on mechanical ventilator support, and 116 (61.1%) patients required vasopressor support upon ICU admission. The median plasma TRAIL level was 34.48 pg/mL in the study patients. While patients on mechanical ventilatory support were more common in the control group, patients requiring vasopressor support were more prevalent in the sepsis group. Furthermore, severity scores of SAPS 3 and SOFA, as well as the C-reactive protein level were higher in the sepsis group. However, no difference in 28-day ICU mortality or in-hospital mortality was observed. The plasma TRAIL level for 143 patients with either sepsis or septic shock was 31.55 pg/mL, which was lower compared to that of 52.00 pg/mL in the control group.

**Figure 2:**
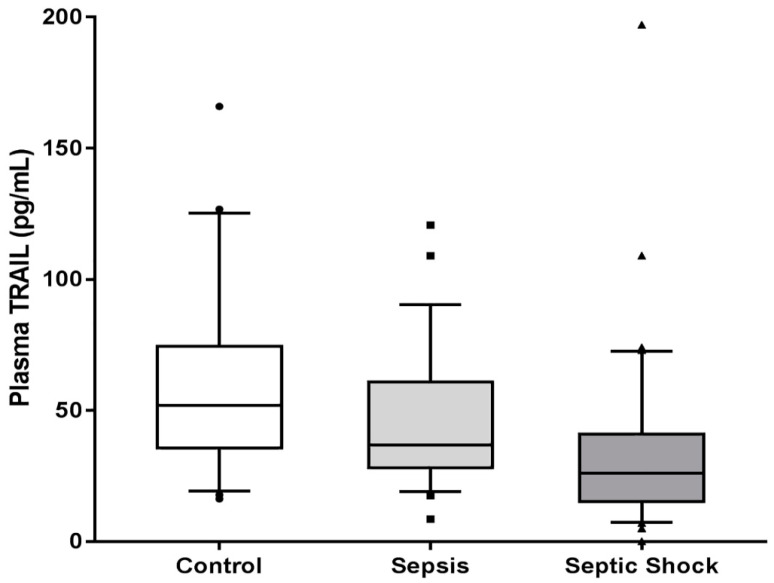
Plasma levels of Tumor necrosis factor TNF-related apoptosis-inducing ligand (TRAIL) in control, sepsis, and septic shock (*p* for trend < 0.001).

**Figure 3:**
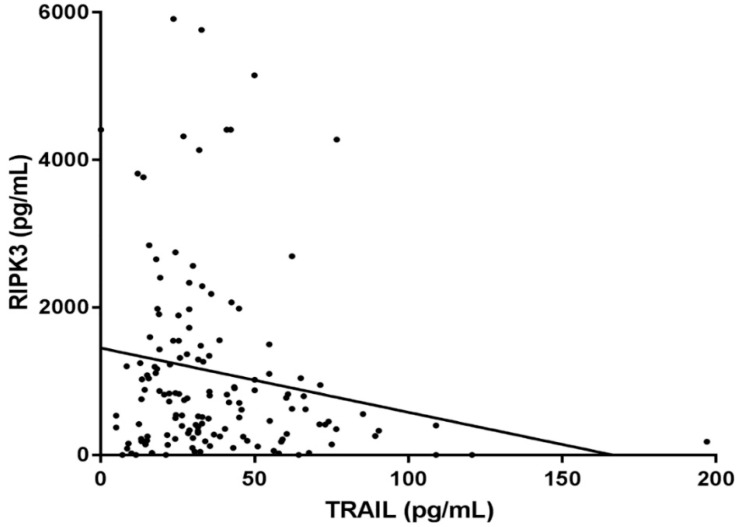
Correlation between plasma level of TRAIL and RIPK3 in patients with sepsis (n = 143). Slope: −8.713 (95% CI: −0.426–−17.000), r^2^: 0.02973, Pearson’s r: −0.172 (*p* = 0.039).

There was a statistically significant trend of decreased plasma TRAIL level across control, sepsis, and septic shock groups (52.00 [35.66–74.41] pg/mL vs. 35.83 [28.07–60.82] pg/mL vs. 26.08 [15.29–40.97] pg/mL; *p* < 0.001) (Figure 2). When correlation between TRAIL and RIPK3 was assessed, the plasma level of TRAIL was inversely related to the plasma level of RIPK3 in patients with sepsis and septic shock (r = −0.172, r^2^ = 0.02973, *p* = 0.039) (Figure 3).

**Figure 4:**
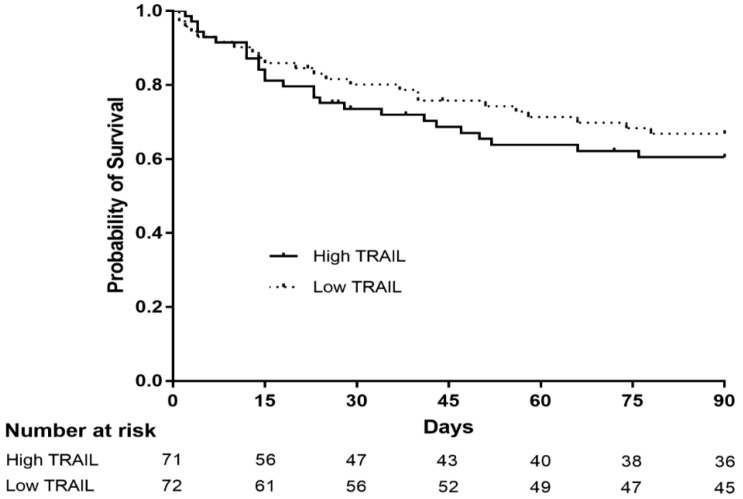
Kaplan–Meier survival analysis comparing patients with sepsis or septic shock with high and low plasma level of TRAIL (n = 143). The 30-day and 90-day survival estimates are 76.6% and 60.5%, respectively for patients with high TRAIL (solid line), while it is 80.2% and 66.9% for patients with low TRAIL (dotted line) (*p* = 0.419, log-rank test).

To evaluate the association between characteristics of patients with sepsis and plasma TRAIL level, 143 patients who were diagnosed with either sepsis or septic shock were divided into two groups—low and high plasma TRAIL level—according to the median TRAIL level (Table 2). Septic shock and patients requiring vasopressor support were more prevalent in the group with low plasma TRAIL. Furthermore, lactic acid, the SAPS 3 score, APACHE II score, and SOFA score at initial ICU admission were significantly higher in patients with low plasma TRAIL. However, statistical differences in 28-day ICU mortality, in-hospital mortality, and 90-day mortality were not observed between the two groups. In addition, Kaplan–Meier survival estimation did not demonstrate a difference in 90-day survival between patients with high and low plasma TRAIL level (*p* = 0.419) (Figure 4).

**Figure 5:**
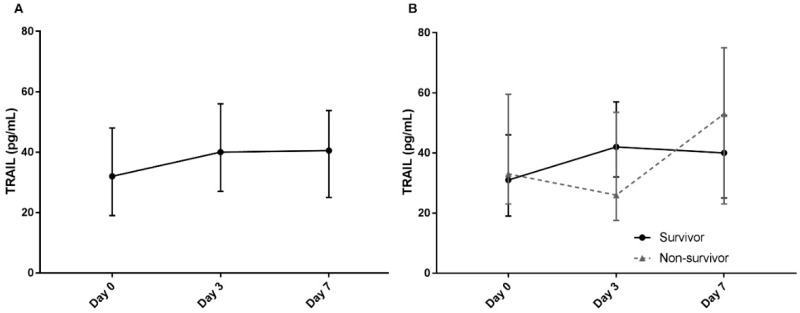
(**A**) Serial plasma levels of TRAIL on days 0, 3, and 7 of 143 patients with sepsis. (**B**) Comparison of serial plasma TRAIL levels in 28-day intensive care unit survivors (solid line) and non-survivors (dotted line). Upper and lower bars represent the 75th and 25th interquartile ranges, respectively.

Finally, serial levels of plasma TRAIL were analyzed in 143 patients with sepsis. Blood samples at days 3 and 7 were available for TRAIL measurement in 59 and 32 patients, respectively. Plasma TRAIL levels increased over time, with median levels of 31.55 pg/mL (19.29–47.56) at day 0, 40.00 pg/mL (27.00–56.00) at day 3, and 40.50 pg/mL (25.00–53.75) at day 7 (*p* < 0.001) (Figure 5A). Although the TRAIL level on day 3 was significantly higher compared to that of day 0 (*p* = 0.036), there was no difference in levels between days 3 and 7. Plasma TRAIL levels were also assessed according to 28-day ICU mortality (Figure 5B). While serial levels of TRAIL significantly increased over time in survivors (*p* = 0.005), TRAIL levels in non-survivors did not demonstrate any differences (*p* = 0.102). The TRAIL level in survivors was higher on day 3 compared to day 0 (*p* = 0.039); however, TRAIL levels of day 3 and day 7 did not differ. Conversely, there was no difference in levels of TRAIL between day 0 and day 3, or between day 3 and day 7 in non-survivors. Finally, when plasma TRAIL levels on days 3 and 7 were compared between survivors and non-survivors, there was a tendency for a higher TRAIL level only on day 3 in survivors compared to non-survivors [42.00 (32.00–57.00) vs. 26.00 (17.50–53.50), *p* = 0.0502]. Nonetheless, the day 7 levels demonstrated no difference between survivors and non-survivors [40.00 (25.00–52.50) vs. 53.00 (23.00–75.00), *p* = 0.801].

## 4. Discussion

The aim of this prospective observational study was to investigate the association between plasma TRAIL level and sepsis severity, as well as to determine the role of TRAIL in predicting mortality. In addition, we planned to analyze the correlation between plasma levels of TRAIL and RIPK3 to identify the potential relationship of TRAIL with necroptosis. Our study of 190 consecutively enrolled critically ill patients demonstrated that the plasma TRAIL level was inversely associated with sepsis severity and plasma level of RIPK3; however, it was not predictive of mortality.

Until now, only two studies have investigated the significance of plasma TRAIL in sepsis. Tian et al. reported that the level of soluble TRAIL was lower in patients with septic shock (defined by an old definition) compared to healthy controls or patients with sepsis; soluble TRAIL was also associated with mortality [12]. Very recently, a multicenter study demonstrated an inverse correlation between the level of circulating TRAIL and both organ dysfunction and RIPK3; [13] however, the association of TRAIL with mortality was portrayed in only two of three cohorts. The inverse relationship between plasma TRAIL level and sepsis severity observed in our study concurs with the results of two previous studies, strengthening the growing evidence of the potential role of TRAIL in sepsis.

The reasons for the inverse relationship between TRAIL and sepsis severity and the role of TRAIL in sepsis pathogenesis are not fully understood. Nonetheless, there are several suggested mechanisms to explain this phenomenon. One possible explanation is based on the anti-inflammatory effect of TRAIL. TRAIL is thought to facilitate and accelerate apoptosis of leukocytes, contributing to resolution of inflammation [20]. Animal studies have shown that TRAIL is associated with improved survival in murine models of infection and autoimmune diseases [21,22,23]. However, this protective anti-inflammatory effect remains controversial. In a study by Unsinger et al. on murine cecal ligation and puncture models, TRAIL-deficient mice were better able to control bacterial infection due to TRAIL-dependent immune suppression and immune unresponsiveness [24,25]. In addition, plasma TRAIL level was measured at the early phase of sepsis in our studies, as well as previous studies. Since TRAIL-dependent apoptosis affects immune suppression and immune unresponsiveness, which plays a role in the relatively later course of sepsis [25], changes in plasma TRAIL level may not be reflective of immune status. Considering those facts, the anti-inflammatory effect of TRAIL may not fully explain its association with the pathogenesis of sepsis. Another possible mechanism is infection clearance by the TRAIL-dependent pathway. In vitro studies have shown that TRAIL contributes to infection control by mediating elimination of infected cells or restriction of viral replication in influenza or encephalomyocarditis viral infection models [26,27]. However, this observation is also conflicting. Cardoso Alves et al. demonstrated in their study that TRAIL depletion resulted in reduced NK-cell mediated antiviral CD8^+^ T cell killing, thereby leading to faster pathogen clearance in a mouse model of viral infection [28]. Moreover, since pathogen clearance by T cell mediated cell death is crucial in viral infection but not in bacterial infection, it may not fully explain the results of our finding. Finally, the capability of TRAIL to initiate necroptosis, a programmed cell death mechanism, may be another possible theory [10]. Although the precise function and effect of necroptosis in sepsis has yet to be elucidated, the detrimental contribution of necroptosis is thought to lie in cell death, leading to local tissue injury and excess release of pro-inflammatory DAMPs. DAMPs stimulate the innate immune response and trigger an inflammatory cascade, resulting in organ damage [15,29,30,31]. The relationship between sepsis and necroptosis has been observed in murine models, as well as in critically ill patients. Recent studies examining the necroptosis regulators of RIPK1, RIPK3, and MLKL in patients with sepsis reported that their levels were associated with disease severity and/or mortality [32,33]. In certain pathologic environments, such as acidic states, TRAIL triggers necroptosis via RIPK1 and RIPK3 to hinder caspase-dependent apoptosis [10,34]. Our study, as well as the study by Schenck et al. [13], illustrated a significant correlation between the plasma levels of TRAIL and RIPK3, proposing the possibility that TRAIL may have an impact on sepsis through the necroptosis pathway. Nonetheless, it should be noted that this correlation between the TRAIL and RIPK3 proteins may not provide direct evidence that TRAIL reflects the process of necroptosis of sepsis. Although we have identified in our preliminary analysis that the plasma level of TRAIL also correlates with that of mitochondrial DNA (a well-known DAMP) in sepsis (Figures S1–S3), supporting the hypothesis that TRAIL is associated with necroptosis, further research to investigate the pathogenesis of TRAIL in sepsis would be reasonable.

Contrary to previous studies, the plasma TRAIL level was not associated with either 28-day ICU mortality or in-hospital mortality in our study. Interestingly, in the study by Schenck et al. [13], the association between the plasma level of TRAIL with mortality was depicted only in two American cohorts of the United States, but not in a Korean cohort. The authors speculated that different baseline characteristics or processes of care might have contributed to this discrepancy. However, definite causes could not be determined. No relationship between TRAIL and mortality was observed in our cohort, in addition to another Korean cohort studied by Schenck et al. [13]. This raises an ethnic difference as one of the many possibilities. Since the mortality of patients with sepsis may be attributable to various factors, further studies are warranted to confirm the relationship between mortality and TRAIL level and to further investigate the reason for the current discrepancy. 

One noteworthy finding of this study is that serial change in plasma TRAIL levels was available for analysis. To our knowledge, this is the first study to provide trends of the plasma TRAIL in sepsis. In particular, two discoveries require attention. First, TRAIL levels increased over time; however, the levels between days 3 and 7 did not differ. Second, when survivors and non-survivors were divided, the plasma TRAIL level on day 3 compared to day 0 was higher in survivors, but not in non-survivors. That there was no difference between levels on days 3 and 7 suggests that TRAIL, or the mechanism underlying the decrease in TRAIL, stabilizes or recovers within the initial days of sepsis. This suggests that TRAIL may be important in the relatively early phase of sepsis. Furthermore, because the TRAIL level on day 3 increased in survivors but not in non-survivors, perhaps it is not only the initial TRAIL level, but also the early serial changes of TRAIL that are crucial in understanding the patient’s course and predicting mortality. However, since the day 3 and 7 samples were available only in some of the 143 patients with sepsis, we could not draw a definitive conclusion. As we have limited understanding of the role of TRAIL in sepsis, changes in TRAIL levels may be a consequence of sepsis or organ failure, rather than demonstration of a key process underlying sepsis. Comprehension of the pathogenesis and kinetics of TRAIL in sepsis is required to determine the significance of serial changes in TRAIL.

To fully appreciate the results of our study, several limitations should be acknowledged. First, our study was conducted at a single referral center, which may limit the generalizability of our data. Second, follow-up data on TRAIL were not available in some patients due to death, recovery, and refusal to collect additional samples. Third, based on our study protocol, enrollment of a study patient and sampling of blood were done within 24 h of ICU admission and 48 h of enrollment, respectively. Therefore, patients with high severity and early mortality may not have been included in the study. Interpretation of results requires caution, since there may be a selection bias. Fourth, the correlation between TRAIL and RIPK3 and its association with severity of sepsis does not provide sufficient evidence that TRAIL functions as an initiator of necroptosis in sepsis or necroptosis is activated in sepsis. Further studies regarding gene expression and quantification are necessary to confirm this relationship.

## 5. Conclusions

In conclusion, plasma TRAIL level was inversely associated with sepsis severity and plasma level of RIPK3. Plasma TRAIL levels increased following the course of recovery. However, they were not predictive of mortality. 

## Figures and Tables

**Table 1:** Baseline characteristics of study patients (n = 190).

Characteristics	Total (N = 190)	Control (n = 47)	Sepsis (n = 143)	*p* Value
Age, years	64 (54–73)	60 (54–68)	67 (53–74)	0.086
Gender, male	121 (63.7)	25 (46.8)	96 (67.1)	0.085
Co-morbidities				
Solid tumor	62 (32.6)	11 (23.4)	51 (35.7)	0.120
Hematologic malignancy	32 (16.8)	7 (14.9)	25 (17.5)	0.681
Diabetes	55 (28.9	12 (25.5)	43 (30.1)	0.552
Chronic obstructive pulmonary disease	18 (9.5)	7 (14.9)	11 (7.7)	0.157
Chronic kidney disease	15 (7.9)	4 (8.5)	11 (7.7)	1.000
Myocardial infarction	11 (5.8)	4 (8.5)	7 (4.9)	0.470
Congestive heart failure	9 (4.7)	4 (8.5)	5 (3.5)	0.228
Cerebrovascular disease	6 (3.2)	0	6 (4.2)	0.339
Chronic liver disease	1 (0.5)	0	1 (0.7)	1.000
Charlson Comorbidity Index	2 (1–3)	2 (1–3)	2 (1–3)	0.554
Clinical status on ICU admission				
Need for MV	95 (50.0)	32 (68.1)	66 (46.2)	0.009
Need for vasopressor support	116 (61.1)	15 (31.9)	101 (70.6)	<0.001
Laboratory findings				
PaO_2_/FiO_2_ ratio	139.3 (125.2–298.1)	255.1 (125.4–362.1)	181.3 (124.7–280.5)	0.112
CRP, mg/dL	10.0 (3.0–19.0)	5.4 (0.8–9.1)	12.5 (4.1–22.5)	<0.001
Lactic acid, mg/dL	3.0 (2.0–4.0)	2.4 (1.2–4.3)	2.7 (1.8–4.0)	0.230
Severity of illness				
SAPS 3, points	52 (44–59)	48 (35–56)	53 (46–59)	0.008
APACHE II score	23 (18–28)	22 (15–27)	23 (19–29)	0.184
SOFA score, initial	8 (5–11)	5 (3–9)	6 (6–11)	<0.001
Plasma TRAIL, pg/mL	34.482 (23.446–56.946)	52.000 (35.657–74.410)	31.545 (19.289–47.564)	<0.001
Outcome				
28-day ICU mortality	39 (20.5)	10 (21.3)	29 (20.3)	0.883
In-hospital mortality	48 (25.3)	12 (25.5)	36 (25.2)	0.961

Note: APACHE II, acute physiology and chronic health evaluation II; CRP, C-reactive protein; ICU, intensive care unit; MV, mechanical ventilation; SAPS 3, simplified acute physiology score 3; SOFA, sequential organ failure assessment; TRAIL, tumor necrosis factor-related apoptosis-inducing ligand.

**Table 2:** Characteristics of patients with sepsis stratified according to the median level of plasma TRAIL (n = 143).

Characteristics	Low TRAIL (n = 72)	High TRAIL (n = 71)	*p* Value
Age, years	67 (55–73)	62 (51–74)	0.418
Gender, male	40 (69.4)	46 (64.8)	0.553
Co-morbidities			
Solid tumor	10 (13.9)	16 (22.5)	0.448
Hematologic malignancy	14 (19.4)	11 (15.5)	0.534
Diabetes	23 (31.9)	20 (28.2)	0.623
Chronic kidney disease	11 (11.6)	7 (7.4)	0.322
Myocardial infarction	8 (11.1)	4 (5.6)	0.217
Congestive heart failure	3 (4.2)	3 (4.2)	1.000
Charlson Comorbidity Index	2 (1–3)	2 (1–3)	0.668
Septic shock	52 (72.2%)	32 (45.1%)	0.001
Clinical status on ICU admission			
Need for MV	36 (50.0)	30 (42.3)	0.353
Need for vasopressor support	61 (84.7)	40 (56.3)	<0.001
Laboratory findings			
PaO_2_/FiO_2_ ratio	191.7 (120.2–282.6)	180.6 (130.5–277.5)	0.436
CRP, mg/dL	13.7 (4.2–13.7)	12.0 (4.0–22.5)	0.305
Lactic acid, mg/dL	3.1 (2.1–4.5)	2.3 (1.7–3.3)	<0.001
Severity of illness			
SAPS 3, points	56 (50–68)	50 (42–56)	<0.001
APACHE II score	24 (19–30)	22 (19–27)	0.007
SOFA score, initial	9 (7–11)	7 (5–11)	<0.001
Outcome			
28-day ICU mortality	12 (16.7)	17 (23.9)	0.279
In-hospital mortality	19 (26.4)	17 (23.9)	0.736
90-day mortality	23 (31.9)	26 (36.6)	0.556

Note: APACHE II, acute physiology and chronic health evaluation II; CRP, C-reactive protein; ICU, intensive care unit; MV, mechanical ventilation; SAPS 3, simplified acute physiology score 3; SOFA, sequential organ failure assessment; TRAIL, tumor necrosis factor-related apoptosis-inducing ligand.

## Supplementary Materials

The following are available online at https://www.mdpi.com/2077-0383/9/6/1661/s1, Association of plasma level of TNF-related apoptosis inducing ligand with mitochondrial DNA in sepsis. Figure S1: Correlation between plasma level of TRAIL and mtDNA in patients with 49 patients with sepsis, Figure S2: Correlation between plasma level of RIPK3 and mtDNA in patients with 49 patients with sepsis, Figure S3: Plasma levels of mtDNA in sepsis and septic shock.

## Abbreviations

APACHE II: Acute physiology, age, chronic health evaluation II; DAMPs: Damage-associated molecular patterns; ICU: Intensive care unit; RIPK3: Receptor-interacting protein kinase-3; SAPS3: Simplified acute physiology score 3; SOFA: Sequential organ failure assessment; TRAIL, Tumor necrosis factor related apoptosis-inducing ligand.
