# Supplementary material for: Association of Plasma Level of TNF-Related Apoptosis-Inducing Ligand with Severity and Outcome of Sepsis
Source: J Clin Med. 2020 Jun 1;9(6):1661. doi: 10.3390/jcm9061661 (PMC7356503; doi:10.3390/jcm9061661)
Supplement: Supplementary file 1 [file jcm-09-01661-s001.pdf]

Supplementary

Association of plasma level of TNF-related apoptosis inducing ligand with mitochondrial DNA in sepsis

Hongseok Yoo, Jin Young Lee, Junseon Park, Jeong Hoon Yang, Gee Young Suh and Kyeongman Jeon

Of 143 patients with sepsis, plasma level of mitochondrial DNA (mtDNA) was measured from plasma samples of 49 patients (26 patients with sepsis without shock and 23 patients with septic shock). Linear regression was applied to assess the association of mtDNA with TRAIL and RIPK3.

Plasma levels of TRAIL and RIPK3 were compared with mtDNA in 49 patients. There was a tendency of an inverse correlation between plasma level of TRAIL and mtDNA (Pearson's r: -0.181, *P* = 0.213) (Figure S1).

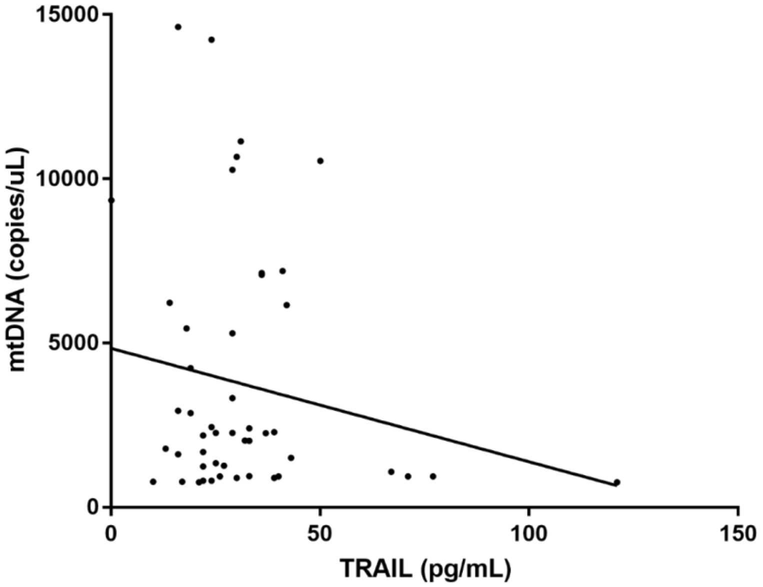

Figure 1. Correlation between plasma level of TRAIL and mtDNA in patients with 49 patients with sepsis. (n = 49) Slope: -6.361 (95% CI: -19.09 – 6.637), r2: 0.033, Pearson's r: -0.181 (P = 0.213).

Regarding RIPK3, there was a significant positive correlation between plasma level of RIPK3 and mtDNA (Pearson's r: 0.693, P < 0.001) (Figure S2).

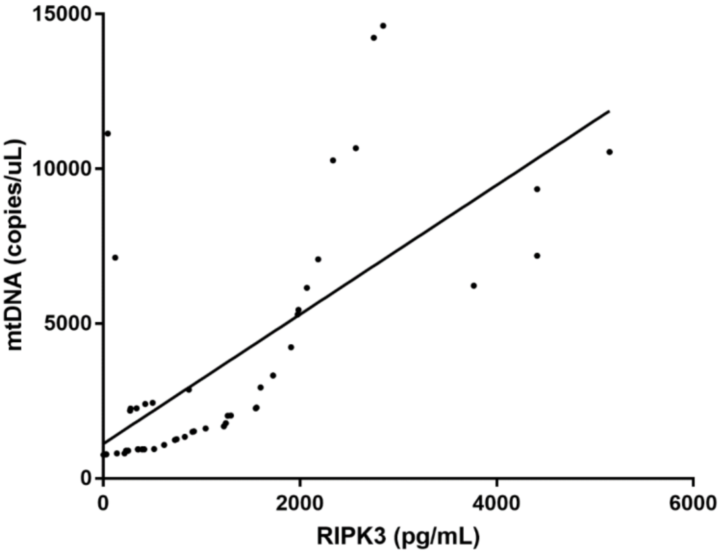

Figure 2. Correlation between plasma level of RIPK3 and mtDNA in patients with 49 patients with sepsis. (n = 49) Slope: 2.089 (95% CI: 1.452 – 2.726), r2: 0.4806, Pearson's r: 0.693 (P < 0.001).

Furthermore, when mtDNA was compared between sepsis and septic shock, plasma level of mtDNA in septic shock patients was higher compared to that in patients without septic shock (Figure S3).

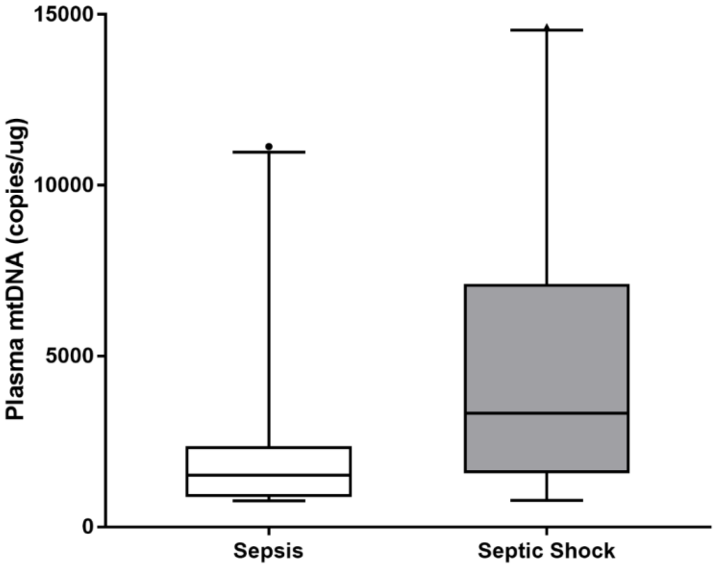

Figure 3. Plasma levels of mtDNA in sepsis and septic shock (Mann-Whitney *P* = 0.0277).
